# Formulation of Emulgels Containing Clotrimazole for the Treatment of Vaginal Candidiasis

**DOI:** 10.3390/gels10110730

**Published:** 2024-11-12

**Authors:** Zsófia Vilimi, Márton Király, Ádám Tibor Barna, Zsófia Edit Pápay, Lívia Budai, Krisztina Ludányi, Nikolett Kállai-Szabó, István Antal

**Affiliations:** 1 Department of Pharmaceutics, Semmelweis University, 1092 Budapest, Hungary; vilimi.zsofia@semmelweis.hu (Z.V.); kiraly.marton@semmelweis.hu (M.K.); barna.adam@semmelweis.hu (Á.T.B.); papay.zsofia@semmelweis.hu (Z.E.P.); budai.livia@semmelweis.hu (L.B.); ludanyi.krisztina@semmelweis.hu (K.L.); kallai.nikolett@semmelweis.hu (N.K.-S.); 2 Center for Pharmacology and Drug Research & Development, Semmelweis University, 1085 Budapest, Hungary

**Keywords:** emulgels, hydrogels, vaginal candidiasis, sustained release, clotrimazole, thermoresponsive hydrogels, vaginal drug delivery system, vaginal gels

## Abstract

Vaginal candidiasis poses significant health concerns that affect approximately 75% of women globally and often leads to discomfort and a decrease in quality of life. Traditional treatments, despite their effectiveness, may cause discomfort and adverse effects, such as vaginal discharge, bleeding, and dryness, promoting the exploration of alternative formulations. In this study, we aimed to develop a novel therapeutic approach for the treatment of vaginal candidiasis utilizing oleic acid containing emulgels made from thermoresponsive poloxamer-based hydrogels. These emulgels were designed to provide a sustained release of clotrimazole, an antifungal agent. Incorporating oleic acid enhanced the drug’s solubility and contributed to vaginal health. The formulations were characterized by their rheological properties, in vitro release, mucoadhesion, and spreadability. We conducted rheological measurements on the hydrogels that served as the base for the emulgels, as well as on the emulgels themselves. The emulgels exhibited continuous rheological behavior with changing temperatures, making them suitable for storage at room temperature. With an increasing HPMC content, we achieved enhanced mucoadhesion, which is beneficial for formulations used in body cavities. Moreover, in vitro release studies revealed sustained drug release profiles, which can be adjusted by varying the ratios of poloxamers and HPMC. These findings suggest that the developed emulgels offer a promising therapeutic option for vaginal candidiasis, addressing both the symptoms and the treatment of discomfort.

## 1. Introduction

Vaginal candidiasis is a widely prevalent issue, affecting approximately 75% of women at least once in their lifetime [1]. Sometimes, recurrent infections impose a significant burden on patients. Several factors can contribute to the development of vaginal candidiasis, including hormonal changes (such as pregnancy, menopause, birth control pills, etc.), stress, the use of different antibiotics, and a weakened immune system. These factors disrupt the delicate balance of vaginal flora, leading to the proliferation of *Candida* species, particularly *Candida albicans*, which is the most common cause [2,3,4]. While vaginal candidiasis may not pose a significant health problem in terms of mortality or morbidity, its symptoms can cause considerable discomfort and greatly diminish the quality of life for women. Recurrent infections can escalate into chronic issues, impacting daily activities and overall well-being [5].

The treatment of these conditions may entail major discomfort beyond the existing symptoms, with unpleasant side effects, like bleeding, vaginal dryness, and itching. This often results in seeking alternative or adjunctive therapies to alleviate symptoms and improve outcomes [6]. Semi-solid preparations for vaginal use, such as creams or gels, have gained popularity among women due to their ease of use, customizable dosage, and the absence of the unpleasant phenomenon of solid excipient reflux. Among semi-solid formulations, hydrogels are particularly prominent and gaining increasing attention [7]. The retention time of conventional formulations is often too short to achieve the desired therapeutical effects; however, mucoadhesive semi-solid formulations have shown therapeutic success due to their spreading ability and their ability to adhere to vaginal mucosa, prolonging contact time and enhancing drug absorption [8].

In this study, to obtain an improved treatment modality, we turned to thermoresponsive hydrogels, which are currently very popular for pharmaceutical formulations. These hydrogels can undergo reversible phase transitions in response to temperature changes, making them particularly suitable for vaginal applications [9,10,11]. In these formulations, we utilized such hydrogels, employing various ratios of poloxamers (PLXs), which are copolymers of poly(ethylene oxide) (PEO) and poly(propylene oxide) (PPO) [12,13]. The popularity of poloxamers is attributed not only to their thermoresponsive properties but also to their low toxicity and high biocompatibility [14]. They serve not only as excellent gelling agents but also as good emulsifiers, facilizing the incorporation of lipophilic active ingredients [15]. Thanks to the properties listed above, they are suitable for creating vaginal formulations while carrying minimal risk.

Clotrimazole (CLZ) was chosen as the model active ingredient. Clotrimazole is a widely used fungistatic agent that inhibits ergosterol synthesis in the fungal cell membrane, altering the permeability of the cell membrane and leading to the destruction of fungal cells. Clotrimazole is a generally well-tolerated substance, but common side effects include skin irritation, itching, burning sensation, and skin rashes [16,17,18]. Its use is usually limited by its low water solubility (0.49 mg/L), but it is much higher in oleic acid, at 180.7 ± 0.04 mg/mL [19,20].

Previously made hydrogels were able to form an emulgel with oleic acid (OA) that already contained dissolved clotrimazole. Oleic acid is a monounsaturated fatty acid that, in addition to functioning as a solvent for clotrimazole in formulations, may also have other beneficial effects, such as hydrating the vagina and maintaining the proper balance of vaginal bacteria [21,22].

To increase the mucoadhesivity and retention time, we also used hydroxypropyl methylcellulose (HPMC), which is commonly used for such purposes. HPMC is a semi-synthetic polymer that has a high capacity to adhere to biological mucous membranes, including vaginal tissue. By promoting adhesion, it allows the active ingredient to exert its effect for a longer period at the desired site [23,24,25].

In this study, we aimed to create a conveniently usable emulgel dosage form that offers a simpler and more comfortable application, ensures more uniform drug delivery, and thus possesses a favorable side effect profile, ultimately enhancing patient compliance. By combining the advantages of mucoadhesive, oil-containing semi-solid formulations with the thermoresponsive properties of hydrogels, we sought to develop a novel therapeutic approach for the management of vaginal candidiasis, addressing not only the symptoms but also the unpleasant side effects.

## 2. Results and Discussion

### 2.1. Physical Appearance of the Gels

**Figure 1:**
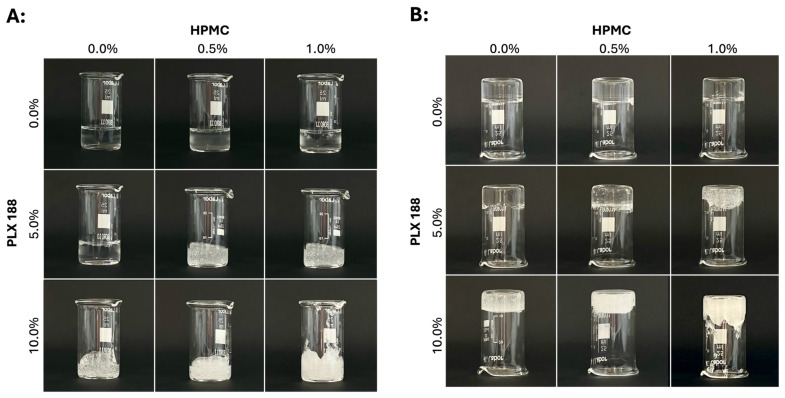
The physical appearance of poloxamer 407-based hydrogels modified with various ratios of excipients at room temperature. (**A**) The formulations in the gel state at room temperature. (**B**) The hydrogels at room temperature in inverted beakers.

The prepared hydrogels exhibited an increasingly viscous flow as the concentration of the gelling excipient increased at 4 °C. The hydrogels with a lower PLX content (the gel containing 21% PLX407, and the gel containing 21% PLX407 and 5% PLX188 but no HPMC) remained transparent, while those with higher concentrations (those that contained PLX407, PLX188, and HPMC) became slightly opalescent. At room temperature, due to the thermoresponsive nature of poloxamer 407, the gels transitioned to a non-flowing gel state (Figure 1).

**Figure 2:**
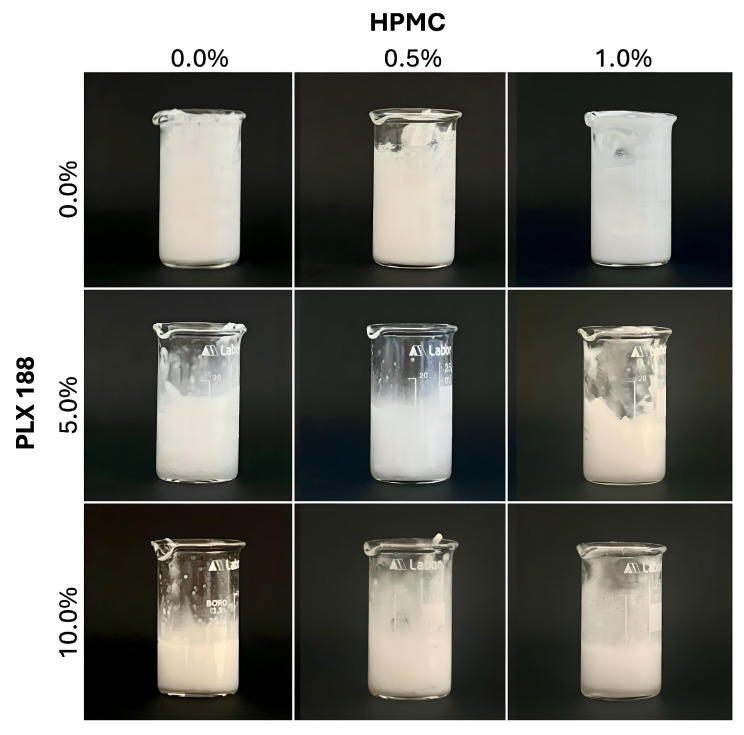
The physical appearance of the emulgels with different gelling agent compositions at room temperature.

When oleic acid was emulsified into them, the hydrogels maintained a creamy/ointment-like consistency, both at room temperature and when stored at lower temperatures (Figure 2).

### 2.2. Rheological Measurements

The examined gels can be categorized into three main groups based on their contents of poloxamer 407 and poloxamer 188, as well as into three groups based on their HPMC content. Firstly, we investigated the effect of increasing the amount of poloxamer 188 on the gelation temperature and the temperature-dependent viscosity. All hydrogels examined, when removed from the refrigerator, appeared colorless, clear, transparent, and slightly more viscous. The hydrogels visibly altered their viscosity rapidly upon transitioning from refrigeration to room temperature. The sol-gel transition temperatures were determined based on the inflection point of the viscosity–temperature curves, as indicated in the table below (Table 1).

**Figure 3:**
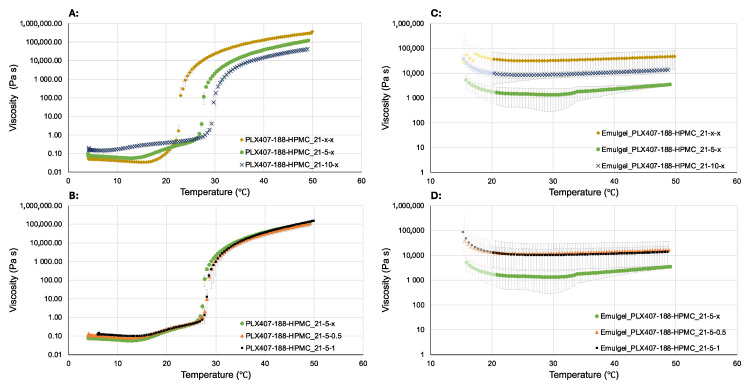
(**A**) Effects of increasing amounts of PLX188 on the gelling temperature of hydrogels; (**B**) effects of increasing amounts of HPMC on the gelling temperature of hydrogels; (**C**) effects of increasing amounts of PLX 188 on the viscosity of emulgels; (**D**) effects of increasing amounts of HPMC on the viscosity of emulgels.

The thermogelling of poloxamer 407 aqueous solutions is a result of the dehydration of the hydrophobic block of the copolymer poly(propylene oxide) [26]. Based on the former, the gelation temperature of a poloxamer aqueous solution can be easily regulated by adding another poloxamer containing different ratios of PEO and PPO blocks. By adding poloxamer 188, the total poloxamer content, and hence, the mechanical stability of the gels is increased; however, the sol-gel transition temperature can be altered by changing the ratio of the PEO and PPO blocks. It was also observed that with an increasing amount of poloxamer 188, the “initial viscosity” measured at low temperatures of the gels increased, but the viscosity values measured at high temperatures decreased compared to the hydrogels without any poloxamer 188 content. Thus, the addition of poloxamer 188 can narrow the range of achievable viscosity. Based on the gelation temperatures indicated in the figure (Figure 3A) and table (Table 1), it can be seen that increasing the amount of poloxamer 188 resulted in a higher sol-gel transition temperature, which is therapeutically more favorable as it approaches the body temperature more closely. HPMC was added to the gels in various percentages to increase their mucoadhesion. Based on the experiments, the amount of HPMC did not significantly influence the viscosity or gelation temperature (Figure 3B). The hydrogels were supplemented with a large amount, 30% *w*/*w*, of oleic acid. The inclusion of oleic acid aids in sustaining the appropriate ratio of vaginal microflora, contributing to the overall vaginal health [21]. Additionally, the high solubility of clotrimazole in OA ensures that the complete amount of clotrimazole required for standard dosage is present in its dissolved form in the final pharmaceutical formulation. However, due to its very high melting point, the handling of oleic acid requires careful management [27]. During the emulsification of oleic acid with the poloxamer hydrogels, we observed an immediate formation of a gel. Consequently, the resulting emulgel formulations exhibited consistent viscosity at both low and high temperatures, which is advantageous for their shelf-life stability (Figure 3C,D). The amount of HPMC did not influence the viscosities greatly; however, the presence of it slightly increased the flowing properties. The measurements conducted at very low temperatures were significantly affected by the high melting point of oleic acid (approximately 12 °C). Since the viscosity of the emulgels remained constant with the temperature variations, there was no sol-gel transition temperature in their case.

### 2.3. In Vitro Release

The drug release from the vertical diffusion cell was determined by normalizing the amount of the released drug to the surface area of the impregnated membrane in mg/cm^2^. The membrane area was 1.77 cm^2^. During the experiments, it was observed that increasing the concentrations of poloxamer 188 and HPMC accelerated the drug release rate. Clotrimazole dissolved in oleic acid exhibited a significantly slow and continuous release rate from our formulations (Table 2).

**Figure 4:**
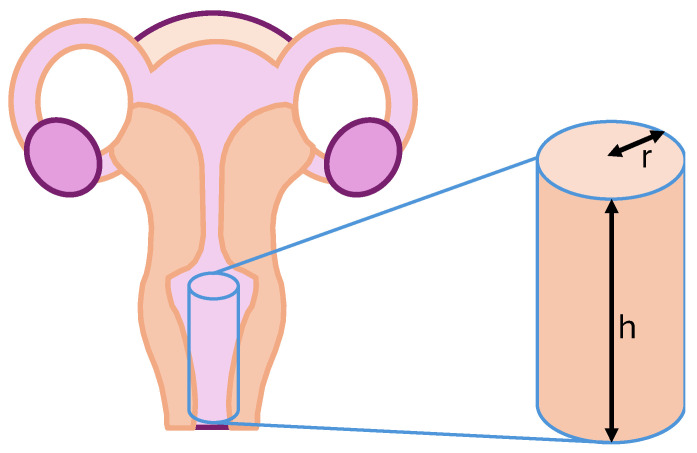
Calculation of average vaginal size. The “r” denotes the average radius of the body cavity, while “h” represents its depth.

Since the amount of drug released per unit surface area does not intuitively convey the results, the measured values were multiplied by the average surface area of the vagina. Surface area calculations were performed based on a model resembling a cylinder open at both ends (Figure 4), with an average depth of 7.57 cm and width of 4.67 cm, resulting in an average surface area of 111.01 cm^2^. However, it should be noted that individual variations in shape and dimension can be highly diverse [28].

**Figure 5:**
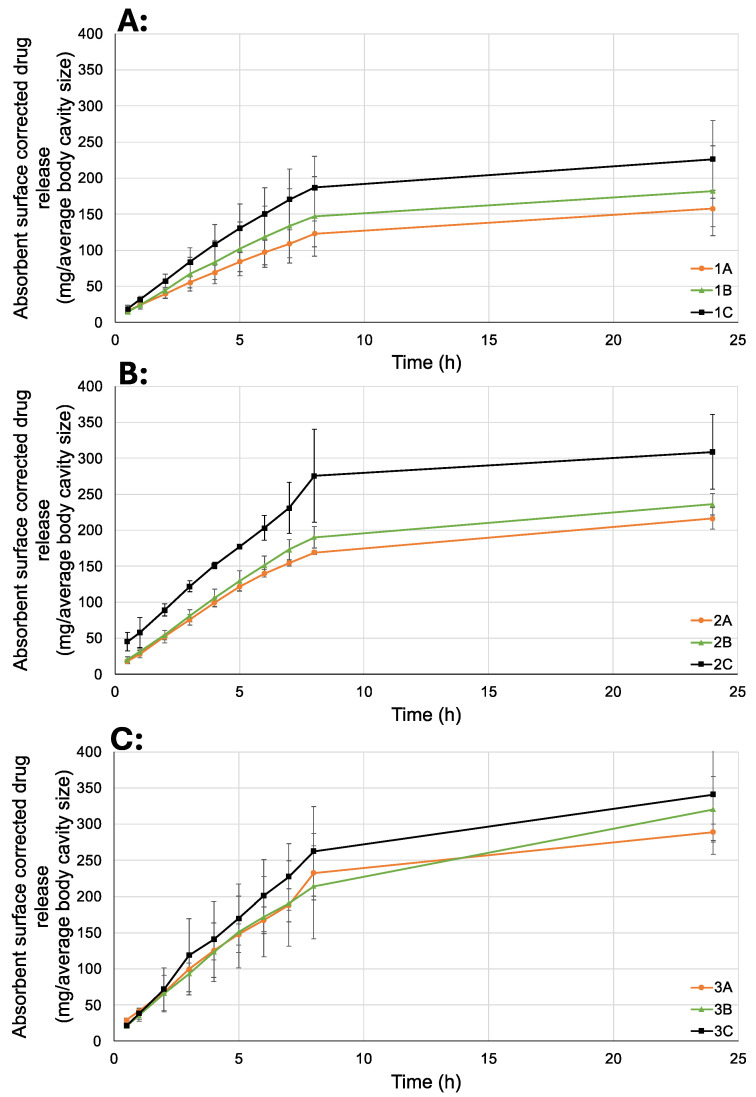
The amounts of released clotrimazole corrected with the average body cavity size; (**A**) samples 1 A–1 C: 0% PLX188; (**B**) samples 2 A–2 C: 5% PLX 188; (**C**) samples 3 A–3 C: 10% PLX 188.

The results of multiplying the measured values by the potential absorptive surface area represent the drug release per square centimeter (Figure 5). Considering the typical vaginal gel products available on the market, approximately 5 g can be inserted into the vagina [29,30]. The amount of clotrimazole incorporated into our formulation was adjusted to match the amount typically inserted into an average vagina, resulting in a formulation containing 200 mg of clotrimazole per 5 g of gel [31,32]. This aligns with the usual single dose of 200 mg of clotrimazole. Taking these factors into account, the prepared formulations may be capable of continuously releasing the entire incorporated amount of the active ingredient over 24 h. The dissolution profile indicates that the formulations may be capable of carrying both the smaller, typical 200 mg doses and larger, single 500 mg doses [33].

**Figure 6:**
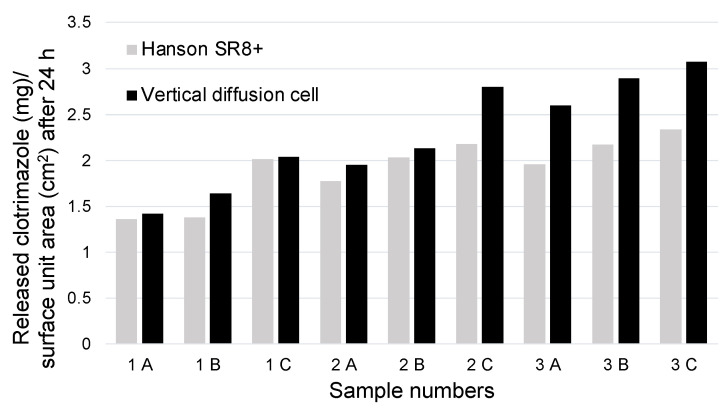
Amounts of active ingredient released per surface unit area over 24 h using different methods. The results of the two methods exhibit the same pattern.

The drug release measurements conducted in the paddle dissolution testing apparatus were also normalized to the surface area of the membrane used. The measured sample quantities, the volume of the dissolution media, and the membrane surface area were bigger compared to the measurements performed in the vertical diffusion cell. The surface area of the membrane was 11.24 cm^2^. The results from the two methods exhibit the same pattern, and the final outcomes are comparable (Figure 6).

The findings of this study confirm the results obtained with the vertical diffusion cell, indicating that increasing the amounts of poloxamer188 and HPMC accelerated the drug release rate. The increased drug release from the gels containing higher amounts (5% and 10%) of poloxamer 188 may be due to its extremely high HLB value, which facilitates the release of poorly water-soluble active substances, such as clotrimazole [34]. The slower release of clotrimazole may also be due to the higher amount of poloxamer 407 present and its pronounced reverse thermogelling properties. At body temperature, poloxamer 407 may form a diffusion barrier gel layer, which delays drug release [35]. Although both HPMC and the poloxamers have gelling properties, they differ in their gel-forming processes. While poloxamers form micelles, HPMC forms gels by swelling. The different water absorption processes can lead to disruption of the gel structure, resulting in faster release of the active ingredient [36,37].

To understand the kinetic release of clotrimazole from the emulsion gels in the first 8 h, we performed an analysis using Higuchi’s model. The correlation coefficients (r) (Table 3) indicate that the fit was good and the model fit well, as the minimum value of r was 0.9847. It can be clearly seen that the concentration of additional polymers (poloxamer 188 and HPMC) to poloxamer 407 in the composition has a strong influence on the slope. Increasing the amounts of HPMC and PLX 188 in the formulation increases the value of the slope, and a faster drug release is expected.

### 2.4. Mucoadhesion Measurement

During the mucoadhesion test conducted with a texture analyzer, the lower tray of the apparatus was covered with porcine mucosa, and equal amounts of the formulations were placed onto this surface. The instrument geometry was immersed in the gels, submerged for 2 s, and then started to be withdrawn. The force required for detachment, i.e., separation from the gel, was recorded during the lifting process. The easier the geometry detached from the gel, the better adhesion the gel exhibited to the porcine mucosa, indicating higher mucoadhesivity properties. Our measurements show that increasing the amount of poloxamer 188 enhanced the spreading ability of the gels, leading to better adhesion to the mucosal surface. The addition of HPMC also clearly increased the mucoadhesion. The gel containing neither poloxamer 188 nor HPMC exhibited the least adhesion (Table 4).

### 2.5. Spreadability

**Figure 7:**
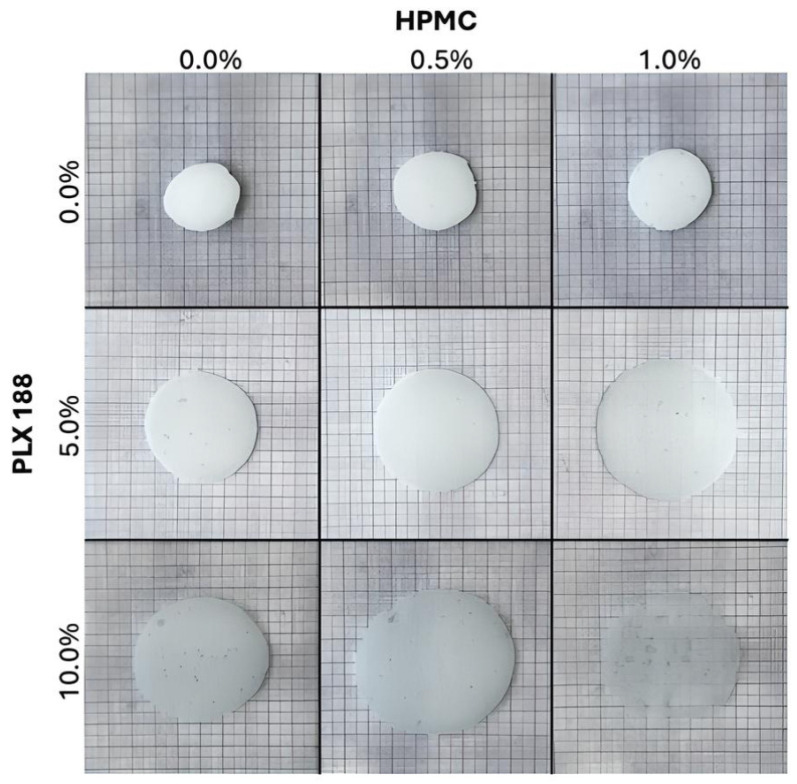
The spreadability of the emulgels with different PLX188 and HPMC contents.

The spreadability tests correlated with the results of the mucoadhesiveness examination. Emulgels with higher PLX188 content visibly covered a larger surface area when placed between the two glass plates (Figure 7). Phase separation was also observed in the gels with the highest PLX188 concentration, raising concerns about their physical stability. The viscosity measurements show that the emulgels containing 5% PLX188 had the lowest viscosity. This could be because phase separation did not occur, but the higher PLX188 content decreased the viscosity of the emulgels by affecting the micelle formation, and thus, the overall gel structure [38,39,40]. At 10% PLX188 content, phase separation became visible, which may have caused the OA to flow away during the geometry’s rotation under the rheological measurement, resulting in more of the ‘hydrogel’ being measured than the emulgel. Formulations with better spreadability values are very likely to cover a larger surface area of the vaginal wall.

### 2.6. Microstructural Investigation

**Figure 8:**
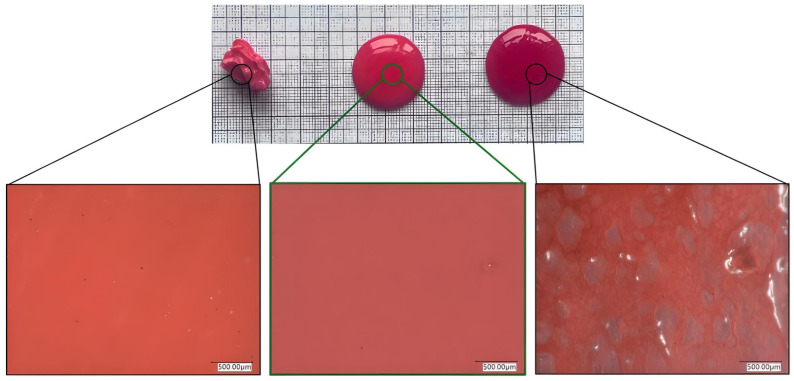
Microscopic examination of emulgels with different compositions at room temperature to assess their stability after 12 h. From left to the right: The first sample contained only poloxamer 407 in 21%. The second sample is the middle sample among those measured, containing 21% poloxamer 407, 5% poloxamer 188, and 0.5% HPMC. In the first two pictures, no oil droplets can be. In the third picture, phase separation can be seen not just under the microscope, but with the naked eye as well. The third sample contains 21% poloxamer 407, 10% poloxamer 188, and 1% HPMC.

To confirm the phase separation, and thus, the stability of the gels, we examined three of the prepared gels (samples 1A, 2B, and 3C) under a microscope (Figure 8). The gel containing only PLX407 was stable, with no phase separation visible under the microscope; however, its consistency was inadequate, as it was difficult to spread based on the spreadability tests, making it unsuitable for coating the body cavities. The emulgel containing 5% PLX188 and 0.5% HPMC was deemed appropriate based on its spreadability, as no phase separation was observed either microscopically or organoleptically. In contrast, the emulgel containing 10% PLX188 and 1% HPMC showed clear phase separation visible to the naked eye, despite the large amount of gelling excipients present, which was also confirmed by microscopic examination.

## 3. Conclusions

The development of effective and patient-friendly treatments for vaginal candidiasis is imperative due to its widespread prevalence and the significant impact it has on women’s quality of life. In this study, we wanted to satisfy these needs by formulating emulgels with incorporated clotrimazole that provide a sustained release, combining the advantages of a semi-solid, mucoadhesive formulation with the favorable properties of the incorporated oil and the thermoresponsive properties of the hydrogels.

Our results demonstrate the advantages of using a thermoresponsive, poloxamer-based hydrogel for vaginal application. Poloxamers, due to their thermoresponsive properties offer the advantages of easy application and prolonged contact with the vaginal mucosa. Moreover, the addition of oleic acid as a vehicle for clotrimazole ensures its solubility and helps maintain the balance of the vaginal microflora, promoting further vaginal health.

The rheological measurements show that increasing the content of poloxamer 188 led to higher sol-gel transition temperatures, which is favorable for therapeutic efficacy as it aligns with the body temperature. The incorporation of HPMC did not affect the sol-gel transition significantly but increased the mucoadhesion, which is crucial for prolonging drug retention in the vaginal cavity. However, the addition of oleic acid produced an emulgel with constant viscosity at all temperatures, which can result in higher stability.

It was further observed that the different poloxamers and HPMC had mutual effects on each other. The different poloxamer compositions were able to form gel networks of varying strengths, which can be attributed to their different molecular weights, differing HLB values, and the varying ratios of PPO and PEO groups. The gel network formed in the formulations was also influenced by the presence of HPMC. Although HPMC can also be used as a gelling agent, the gelling processes of poloxamers and HPMC differ. While HPMC swells, poloxamers form micelles. As a result, these gelling agents compete for the water present in the formulation or the environment, weakening the consistency of the formulation.

The in vitro release studies demonstrated sustained release of the clotrimazole from the emulgels. The release profile can be adjusted by the ratio of the poloxamers relative to each other. A higher proportion of poloxamer 407 resulted in a slower drug release. This sustained release profile is advantageous compared to commercially available products, potentially leading to improved patient compliance and therapeutic outcomes.

In conclusion, the development of emulgels incorporating clotrimazole in a dissolved form offers a promising therapeutic approach for the treatment of vaginal candidiasis. By combining the advantages of mucoadhesive, oil-containing semi-solid formulations with thermoresponsive hydrogels, our formulations aim to provide sustained release and increased patient compliance.

## 4. Materials and Methods

### 4.1. Materials

Poloxamer 407 (Pluronic F-128; PLX407) and poloxamer 188 (Kolliphor P188; PLX188) were obtained from Sigma-Aldrich (St. Louis, MO, USA). Oleic acid (OA) and hydroypropyl methylcellulose (HPMC) Pharmacoat 606 were purchased from (Shin-Etsu Chemical Co., Ltd.; Tokyo, Japan). Clotrimazole, the broad-spectrum antifungal model active ingredient was procured from Molar Chemicals Kft. (Halásztelek, Hungary). Porcine mucosa, which serves as a model for human mucosal tissues in in vitro studies, was freshly obtained from a local butcher shop. Sudan III was also obtained from Sigma-Aldrich.

### 4.2. Methods

#### 4.2.1. Preparation of Gels

The hydrogels were prepared using the cold method [41].The pre-measured and homogenized PLXs and HPMC were slowly added to re-cooled water at 4–5 °C and then stored in a refrigerator for 48 h. Every 12 h, the mixture was stirred at 150 rpm in a cold environment for 15 min each time to achieve complete dissolution. Depending on the concentration of the gelling agent, we obtained transparent or slightly opalescent gels.

For the preparation of the emulgels, the necessary amount of CLZ was first dissolved in a given amount of oleic acid. The dissolution process was assisted by magnetic stirring at 100 rpm for 2 h. Then, the clotrimazole-containing oleic acid was added gradually to the prepared hydrogels in a cold environment. Homogenization was facilized using an IKA-WERK Ultra-Turrax (IKA-WERK GmbH & Co. KG; Staufen, Germany) for 2 min at 3000 rpm, with 15 min intervals of rest per minute, while applying the gel’s re-cooling. The table below summarizes the exact composition of the formulas produced (Table 5).

#### 4.2.2. Rheological Measurements

To determine the sol-gel transition temperature and the temperature-dependent viscosity of the hydrogels, we used a Kinexus Pro+ rheometer Model KNX2100 (Malvern Instruments Ltd., Malvern, UK). The rSpace software version 1.3 from Kinexus Pro (Netzsch, Bayern, Germany) was employed to record the collected data. The machine was used in the rotating mode with a cone-plate geometry (CP4/40 SR0207 SS: PL65 S0815 SS) with a gap of 0.15 mm. The measurement temperature range was set between 5 and 50 °C. A constant shear stress of 1 Pa was applied during the measurement. Then, 1 g samples were measured on the plate.

To examine the effect of the oleic acid added to the hydrogels and measure the temperature-dependent viscosity, we used the same equipment and conditions; the temperature range was set between 5 and 50 °C, and a constant shear stress of 1 Pa was used. All measurements were performed with three replicates.

#### 4.2.3. In Vitro Release

For the in vitro release measurements, two different methods were employed and compared.

The first method utilized a vertical diffusion cell for drug release assessment. The release medium consisted of 65% pH 4.5 phosphate buffer + 35% ethanol due to the poor water solubility of clotrimazole [17,42,43,44,45]. The volume of the release medium was 7.5 mL, maintained at T = 37 ± 0.5 °C with agitation at 75 rpm. The samples were separated from the medium by a cellulose acetate membrane impregnated with the release medium [46]. The sampling occurred initially after half an hour, followed by hourly intervals for 8 h with replacement of the medium (*n* = 3). Additionally, a sample was taken after 24 h. For analysis of the drug release, the vertical diffusion cell method was performed using UV-Vis spectroscopy following HPLC separation.

The chromatographic separations were performed on an Agilent Series 1100 LC system (Agilent Technologies, Santa Clara, CA, USA). The analytes were separated on a Phenomenex^®^ Luna C18(2) (Torrance, CA, USA) with a 100 Å column (4.6 mm × 250 mm, 5 μm). The column and the autosampler were maintained at 25 °C, and 5 μL of sample was eluted under isocratic conditions over 4 min at a flow rate of 2 mL/min. The mobile phase was composed of water acetonitrile (30:70, *v*/*v*%). The detection was carried out with a UV-DAD detector (Agilent Technologies, Santa Clara, CA, USA) at 210 nm.

The amount of the substance was calculated by applying a predetermined calibration curve. The calibration standard was diluted from the stock solution to obtain seven calibration levels, which were ran in duplicate at the beginning of the measurement process. The linearity value for the clotrimazole was R^2^ = 0.9997. The lowest and highest points of the calibration curve coincided with the lower limit of quantitation (LLOQ) and the upper limit of quantitation (ULOQ). Intra-day accuracy and precision were assessed by evaluating five replicates of one low and one high concentrated QC sample (*n* = 5–5). The accuracy was expressed as a percentage of the nominal concentration, and the precision was calculated as the relative standard deviation (RSD). The acceptance criteria for both parameters were set at ±5%.

In the second method, the gels were loaded into a metal ointment cell immersed in the release medium. The gels were separated from the release medium by a cellulose acetate membrane. The 500 mL of release medium was maintained at T = 37 ± 0.5 °C, consisting of 65% pH 4.5 phosphate buffer +35% ethanol [47,48]. Release studies (*n* = 3) were conducted using a Hanson SR8 Plus (Hanson Research, Chatsworth, CA, USA) dissolution testing apparatus for the paddle method at 50 rpm, with sampling at the time points of 0.5, 1, 2, 3, 4, 5, 6, 7, 8, and 24 h. A sample volume of 5 mL was withdrawn and analyzed spectrophotometrically at a wavelength of 205 nm [49]. The volume of the release medium was replenished after each sampling event.

The Higuchi model was used to study the drug release kinetics of different clotrimazole containing emulgels:

*Q* = *K_H_* ×√*t* (1)

where *Q* is the amount of drug released (mg/area) per unit time, *K_H_* is the Higuchi constant (mg/area × h^−0.5^), and *t* is the time (h). For the fitting, we used the average data from the first 8 h of the dissolution test.

#### 4.2.4. Mucoadhesion Measurement

The mucoadhesive tests were conducted using the CT3-4500 texture analyzer (AMETEK Brookfield, Middleboro, MA, USA). Porcine mucosa was attached to the lower plate of the texture analyzer, onto which an equal amount of gel (1 g) was spread. A cylindrical geometry (TA5 cylinder; 12.7 mm D; 35 mm L) was immersed into the gels, and then lifted from the gel to measure the force required for detachment. The measurements were conducted at room temperature [50,51,52,53].

#### 4.2.5. Spreadability

During the spreadability studies, we assessed the spreading ability of 1 g of formulation by placing the gel between two glass plates of the same size. The weight of the glass placed on top was 120 g. The spreading area was measured after 1 min. The results represent the average of three parallel measurements (*n* = 3). The measurements were conducted at room temperature [8].

#### 4.2.6. Microstructural Investigation

Among the emulgels, we prepared three by staining the oil phase with Sudan III dye. The resulting emulgels were examined using a Keyence digital microscope (Keyence Corporation, Osaka, Japan) at room temperature to gather information about the stability of the gels with different compositions.

#### 4.2.7. Statistical Statement

Data analysis was conducted using Microsoft Excel. The rheological measurements were extracted and processed through Malvern Kinexus Pro+ rheometer software (version 2.0.0.0) for subsequent statistical evaluation. Descriptive statistics were applied to summarize the mean values and standard deviations.

## Figures and Tables

**Table 1:** Sol-gel transition temperatures of the different hydrogels.

PLX188 (%)	0.0			5.0			10.0		
HPMC (%)	0.0	0.5	1.0	0.0	0.5	1.0	0.0	0.5	1.0
Sol-gel transition T (°C)	23.01	26.13	21.71	27.74	28.19	27.82	29.76	30.50	30.90

**Table 2:** Amounts of active substance released per unit area.

Sample	Time (h)									
	0.5	1	2	3	4	5	6	7	8	24
1 A	0.15 ± 0.04	0.22 ± 0.05	0.36 ± 0.06	0.49 ± 0.07	0.62 ± 0.09	0.76 ± 0.12	0.88 ± 0.15	0.98 ± 0.17	1.11 ± 0.16	1.42 ± 0.13
1 B	0.13 ± 0.02	0.22 ± 0.02	0.40 ± 0.09	0.60 ± 0.21	0.75 ± 0.27	0.92 ± 0.34	1.07 ± 0.38	1.21 ± 0.46	1.32 ± 0.50	1.64 ± 0.56
1 C	0.17 ± 0.04	0.29 ± 0.04	0.52 ± 0.08	0.76 ± 0.18	0.97 ± 0.24	1.17 ± 0.30	1.35 ± 0.33	1.53 ± 0.38	1.69 ± 0.38	2.04 ± 0.48
2 A	0.16 ± 0.03	0.25 ± 0.05	0.47 ± 0.08	0.68 ± 0.07	0.90 ± 0.06	1.09 ± 0.05	1.26 ± 0.05	1.39 ± 0.04	1.52 ± 0.02	1.95 ± 0.14
2 B	0.18 ± 0.04	0.28 ± 0.04	0.49 ± 0.06	0.73 ± 0.08	0.96 ± 0.11	1.17 ± 0.13	1.36 ± 0.12	1.56 ± 0.13	1.71 ± 0.14	2.13 ± 0.13
2 C	0.41 ± 0.12	0.64 ± 0.40	0.97 ± 0.60	1.23 ± 0.85	1.50 ± 1.13	1.70 ± 1.30	1.96 ± 1.46	2.20 ± 1.57	2.77 ± 1.82	2.80 ± 0.47
3 A	0.26 ± 0.03	0.38 ± 0.02	0.61 ± 0.04	0.90 ± 0.08	1.13 ± 0.11	1.33 ± 0.13	1.51 ± 0.16	1.70 ± 0.21	2.10 ± 0.34	2.60 ± 0.10
3 B	0.18 ± 0.01	0.32 ± 0.07	0.59 ± 0.23	0.84 ± 0.26	1.11 ± 0.36	1.36 ± 0.45	1.55 ± 0.50	1.71 ± 0.53	1.93 ± 0.65	2.89 ± 0.41
3 C	0.19 ± 0.00	0.34 ± 0.07	0.65 ± 0.27	1.07 ± 0.46	1.27 ± 0.47	1.53 ± 0.43	1.82 ± 0.45	2.05 ± 0.41	2.37 ± 0.56	3.07 ± 0.74

**Table 3:** The results of kinetic analysis of the dissolution data using the Higuchi model.

Parameter	1 A	1 B	1 C	2 A	2 B	2 C	3 A	3 B	3 C
K_H_	38.012	45.697	58.297	53.294	58.385	82.579	67.834	66.286	78.351
Correlation coefficient (r)	0.9907	0.9876	0.9881	0.9882	0.9862	0.9917	0.9882	0.9877	0.9847

**Table 4:** Force required to detach from the surface (g).

PLX 188	0.0			5.0			10.0		
HPMC	0.0	0.5	1.0	0.0	0.5	1.0	0.0	0.5	1.0
Force (g)	71.17 ± 7.28	9.33 ± 0.76	5.67 ± 0.29	7.00 ± 1.32	5.83 ± 0.29	4.25 ± 0.35	5.67 ± 0.76	5.50 ± 0.50	4.83 ± 1.75

**Table 5:** Composition of samples.

Name of the Sample	PLX407 (%; *w*/*w*)	PLX188 (%; *w*/*w*)	HPMC (%; *w*/*w*)	CLZ (%; *w*/*w*)
1 A	21	-	-	4
1 B	21	-	0.5	4
1 C	21	-	1.0	4
2 A	21	5	-	4
2 B	21	5	0.5	4
2 C	21	5	1.0	4
3 A	21	10	-	4
3 B	21	10	0.5	4
3 C	21	10	1.0	4

## Data Availability

The data presented in this study are openly available in article.

## References

[1] InformedHealth.org Cologne, Germany: Institute for Quality and Efficiency in Health Care (IQWiG); 2006–Overview: Vaginal Yeast Infection (Thrush) [Updated 4 April 2022]. https://www.ncbi.nlm.nih.gov/books/NBK543220/.

[2] Grigoriou O., Baka S., Makrakis E., Hassiakos D., Kapparos G., Kouskouni E. (2006). Prevalence of clinical vaginal candidiasis in a university hospital and possible risk factors. Eur. J. Obstet. Gynecol. Reprod. Biol..

[3] Zeng X., Zhang Y., Zhang T., Xue Y., Xu H., An R. (2018). Risk Factors of Vulvovaginal Candidiasis among Women of Reproductive Age in Xi’an: A Cross-Sectional Study. BioMed Res. Int..

[4] Sobel J.D. (2016). Recurrent vulvovaginal candidiasis. Am. J. Obstet. Gynecol..

[5] Donders G., Sziller I.O., Paavonen J., Hay P., de Seta F., Bohbot J.M., Kotarski J., Vives J.A., Szabo B., Cepuliené R. (2022). Management of recurrent vulvovaginal candidosis: Narrative review of the literature and European expert panel opinion. Front. Cell Infect. Microbiol..

[6] Osmałek T., Froelich A., Jadach B., Tatarek A., Gadziński P., Falana A., Gralińska K., Ekert M., Puri V., Wrotyńska-Barczyńska J. (2021). Recent Advances in Polymer-Based Vaginal Drug Delivery Systems. Pharmaceutics.

[7] Haque S.N., Bhuyan M.M., Jeong J.-H. (2024). Radiation-Induced Hydrogel for Water Treatment. Gels.

[8] Ochoa Andrade A., Parente E., Ares G. (2014). Screening of mucoadhesive vaginal gel formulations. Braz. J. Pharm. Sci..

[9] Chatterjee S., Hui P.C., Kan C.-W. (2018). Thermoresponsive Hydrogels and Their Biomedical Applications: Special Insight into Their Applications in Textile Based Transdermal Therapy. Polymers.

[10] Permana A.D., Asri R.M., Amir M.N., Himawan A., Arjuna A., Juniarti N., Utami R.N., Mardikasari S.A. (2023). Development of Thermoresponsive Hydrogels with Mucoadhesion Properties Loaded with Metronidazole Gel-Flakes for Improved Bacterial Vaginosis Treatment. Pharmaceutics.

[11] Argenta D.F., Bernardo B.d.C., Chamorro A.F., Matos P.R., Caon T. (2021). Thermosensitive hydrogels for vaginal delivery of secnidazole as an approach to overcome the systemic side-effects of oral preparations. Eur. J. Pharm. Sci..

[12] da Silva J.B., Cook M.T., Bruschi M.L. (2020). Thermoresponsive systems composed of poloxamer 407 and HPMC or NaCMC: Mechanical, rheological and sol-gel transition analysis. Carbohydr. Polym..

[13] Zarrintaj P., Ramsey J.D., Samadi A., Atoufi Z., Yazdi M.K., Ganjali M.R., Amirabad L.M., Zangene E., Farokhi M., Formela K. (2020). Poloxamer: A versatile tri-block copolymer for biomedical applications. Acta Biomater..

[14] Singh-Joy S.D., McLain V.C. (2008). Safety assessment of poloxamers 101, 105, 108, 122, 123, 124, 181, 182, 183, 184, 185, 188, 212, 215, 217, 231, 234, 235, 237, 238, 282, 284, 288, 331, 333, 334, 335, 338, 401, 402, 403, and 407, poloxamer 105 benzoate, and poloxamer 182 dibenzoate as used in cosmetics. Int. J. Toxicol..

[15] Krstonošić V.S., Sazdanić D.B., Ćirin D.M., Nikolić I.R., Hadnađev M.S., Atanacković Krstonošić M.T. (2024). Characterization of Oil-in-Water Emulsions Prepared with Triblock Copolymer Poloxamer 407 and Low-Molecular-Mass Surfactant Mixtures as Carriers of Grape Pomace Waste Polyphenols. Pharmaceutics.

[16] Dinte E., Iovanov R.I., Bodoki A.E., Colosi I.A., Colosi H.A., Tosa N., Vostinaru O., Tomuta I. (2023). Optimization of a Mucoadhesive Vaginal Gel Containing Clotrimazole Using a D-Optimal Experimental Design and Multivariate Analysis. Polymers.

[17] Nematpour N., Moradipour P., Zangeneh M.M., Arkan E., Abdoli M., Behbood L. (2020). The application of nanomaterial science in the formulation a novel antibiotic: Assessment of the antifungal properties of mucoadhesive clotrimazole loaded nanofiber versus vaginal films. Mater. Sci. Eng. C.

[18] https://www.webmd.com/drugs/2/drug-5115/clotrimazole-vaginal/details.

[19] Samrudhi P., Ravikumar P. (2016). Honey based clotrimazole microemulsion for topical delivery. Indo Am. J. Pharm. Res..

[20] Balata G., Mahdi M., Bakera R.A. (2011). Improvement of solubility and dissolution properties of clotrimazole by solid dispersions and inclusion complexes. Indian J. Pharm. Sci..

[21] Zhu M., Frank M.W., Radka C.D., Jeanfavre S., Xu J., Tse M.W., Pacheco J.A., Kim J.S., Pierce K., Deik A. (2024). Vaginal Lactobacillus fatty acid response mechanisms reveal a metabolite-targeted strategy for bacterial vaginosis treatment. Cell.

[22] https://www.broadinstitute.org/news/common-fatty-acid-may-help-restore-healthy-vaginal-bacteria-after-infection.

[23] Notario-Pérez F., Martín-Illana A., Cazorla-Luna R., Ruiz-Caro R., Bedoya L.-M., Peña J., Veiga M.-D. (2019). Development of mucoadhesive vaginal films based on HPMC and zein as novel formulations to prevent sexual transmission of HIV. Int. J. Pharm..

[24] Aka-Any-Grah A., Bouchemal K., Koffi A., Agnely F., Zhang M., Djabourov M., Ponchel G. (2010). Formulation of mucoadhesive vaginal hydrogels insensitive to dilution with vaginal fluids. Eur. J. Pharm. Biopharm..

[25] Abidin I.Z., Murphy E.J., Fehrenbach G.W., Rezoagli E., Gately N., Major I. (2023). A systematic review of mucoadhesive vaginal tablet testing. Drug Target Insights.

[26] Fakhari A., Corcoran M., Schwarz A. (2017). Thermogelling properties of purified poloxamer 407. Heliyon.

[27] Britannica, The Editors of Encyclopaedia “Oleic Acid”. Encyclopedia Britannica, 8 August 2024. https://www.britannica.com/science/oleic-acid.

[28] Pendergrass P.B., Reeves C.A., Belovicz M.W., Molter D.J., White J.H. (1996). The shape and dimensions of the human vagina as seen in three-dimensional vinyl polysiloxane casts. Gynecol. Obstet. Investig..

[29] https://www.drugs.com/pro/metrogel-vaginal.html.

[30] https://ae.betadine.global/en/ae/product-category/product/feminine-care/betadine-vaginal-gel.

[31] https://www.drugs.com/dosage/clotrimazole-topical.html.

[32] https://www.canesten.hu/ismerje-meg-termekeinket/canesten-huvelytabletta.

[33] del Palacio A., Sanz F., Garcia-Bravo M., Gimeno C., Cuetara S., Miranda P., R-Noriega A. (1991). Single dose treatment of vaginal candidosis: Randomised comparison of amorolfine (50 mg and 100 mg) and clotrimazole (500 mg) in patients with vulvovaginal candidosis. Mycoses.

[34] Alexandridis P., Alan Hatton T. (1995). Poly(ethylene oxide) poly(propylene oxide) poly(ethylene oxide) block copolymer surfactants in aqueous solutions and at interfaces: Thermodynamics, structure, dynamics, and modeling. Colloids Surf. A Physicochem. Eng. Asp..

[35] Kolašinac N., Kachrimanis K., Homšek I., Grujić B., Đurić Z., Ibrić S. (2012). Solubility enhancement of desloratadine by solid dispersion in poloxamers. Int. J. Pharm..

[36] Shriky B., Vigato A.A., Sepulveda A.F., Machado I.P., de Araujo D.R. (2023). Poloxamer-based nanogels as delivery systems: How structural requirements can drive their biological performance?. Biophys. Rev..

[37] Joshi S.C., Chen B. (2009). Swelling, Dissolution and Disintegration of HPMC in Aqueous Media. Proceedings of the 13th International Conference on Biomedical Engineering.

[38] Choi H.-G., Jung J.-H., Ryu J.-M., Yoon S.-J., Oh Y.-K., Kim C.-K. (1998). Development of in situ-gelling and mucoadhesive acetaminophen liquid suppository. Int. J. Pharm..

[39] Chen J., Zhou R., Li L., Li B., Zhang X., Su J. (2013). Mechanical, Rheological and Release Behaviors of a Poloxamer 407/ Poloxamer 188/Carbopol 940 Thermosensitive Composite Hydrogel. Molecules.

[40] Giuliano E., Paolino D., Fresta M., Cosco D. (2018). Mucosal Applications of Poloxamer 407-Based Hydrogels: An Overview. Pharmaceutics.

[41] Brambilla E., Locarno S., Gallo S., Orsini F., Pini C., Farronato M., Thomaz D.V., Lenardi C., Piazzoni M., Tartaglia G. (2022). Poloxamer-Based Hydrogel as Drug Delivery System: How Polymeric Excipients Influence the Chemical-Physical Properties. Polymers.

[42] Souto E.B., Müller R. (2007). Rheological and in vitro release behaviour of clotrimazole-containing aqueous SLN dispersions and commercial creams. Die Pharmazie.

[43] Nawaz A., Jan S.U., Khan N.R., Hussain A., Khan G.M. (2013). Formulation and in vitro evaluation of clotrimazole gel containing almond oil and tween 80 as penetration enhancer for topical application. Pak. J. Pharm. Sci..

[44] El-Houssieny B.M., Hamouda H.M. (2010). Formulation and evaluation of clotrimazole from pluronic F127 gels. Drug Discov. Ther..

[45] Huang J., Jacobsen J., Larsen S.W., Genina N., van de Weert M., Müllertz A., Nielsen H.M., Mu H. (2020). Graphene oxide as a functional excipient in buccal films for delivery of clotrimazole: Effect of molecular interactions on drug release and antifungal activity in vitro. Int. J. Pharm..

[46] de Lima J.A., Paines T.C., Motta M.H., Weber W.B., dos Santos S.S., Cruz L., da Silva C.d.B. (2017). Novel Pemulen/Pullulan blended hydrogel containing clotrimazole-loaded cationic nanocapsules: Evaluation of mucoadhesion and vaginal permeation. Mater. Sci. Eng. C.

[47] Bachhav Y.G., Patravale V.B. (2009). Microemulsion-Based Vaginal Gel of Clotrimazole: Formulation, In Vitro Evaluation, and Stability Studies. AAPS PharmSciTech.

[48] Bhat S.R., Shivakumar H.G. (2010). Bioadhesive controlled release clotrimazole vaginal tablets. Trop. J. Pharm. Res..

[49] Ekiert R.J., Krzek J. (2009). Determination of azole antifungal medicines using zero-order and derivative UV spectrophotometry. Acta Pol. Pharm..

[50] Kast C.E., Valenta C., Leopold M., Bernkop-Schnürch A. (2002). Design and in vitro evaluation of a novel bioadhesive vaginal drug delivery system for clotrimazole. J. Control. Release.

[51] Nho Y.-C., Park J.-S., Lim Y.-M. (2014). Preparation of Poly(acrylic acid) Hydrogel by Radiation Crosslinking and Its Application for Mucoadhesives. Polymers.

[52] Singh V.K., Sagiri S., Khade S., Bhattacharya M.K., Pal K. (2014). Development and characterization of gelatin–polysaccharide based phase-separated hydrogels for prevention of sexually transmitted diseases. J. Appl. Polym. Sci..

[53] das Neves J., Amaral M.H., Bahia M.F. (2008). Performance of an in vitro mucoadhesion testing method for vaginal semisolids: Influence of different testing conditions and instrumental parameters. Eur. J. Pharm. Biopharm..

